# One-Step Affinity Purification of MarathonRT Reverse Transcriptase for RNA Sequencing Applications

**DOI:** 10.21769/BioProtoc.5703

**Published:** 2026-06-20

**Authors:** Jenni K. Pedor, Pavlina Gregorova, Salla M. Kalaniemi, L. Peter Sarin

**Affiliations:** 1 RNAcious Laboratory, Department of Molecular and Integrative Biosciences, Faculty of Biological and Environmental Sciences, University of Helsinki, Helsinki, Finland; 2 Doctoral Programme in Integrative Life Science, University of Helsinki Doctoral School, Helsinki, Finland; 3 HiLIFE Helsinki Institute of Life Science, University of Helsinki, Helsinki, Finland

**Keywords:** MarathonRT, Next-generation reverse transcriptase, Reverse transcription, Enzyme purification, tRNA, RNA-seq

## Abstract

Transfer RNAs (tRNAs) are important regulators of translation and cellular function. Several high-throughput sequencing methods have been developed to quantitatively analyze tRNA isoacceptors in cells. However, the strong secondary structures and extensive post-transcriptional modification of most tRNA molecules present significant challenges for many reverse transcriptases, negatively impacting sequencing library preparation and causing quantification biases. Currently, the field utilizes processive next-generation reverse transcriptases (ngRTs), such as Induro (New England Biolabs) and UltraMarathonRT (RNAConnect), to address these issues. Despite being used in multiple protocols, these commercial products face little competition and remain costly. However, non-commercial alternatives, such as the original MarathonRT (MRT), are available from gene repositories. MRT is a next-generation reverse transcriptase derived from the *Eubacterium rectale* group II intron maturase, which can read through RNA secondary structures and chemical modifications. Here, we present a simplified expression and purification protocol for producing highly active MRT that is stable over 1 year. This cost-effective protocol yields a heterogeneous protein preparation with no discernible competing enzymatic activities; it mitigates previously reported precipitation issues, saving one day of laboratory work and eliminating two chromatography-based purification steps. Moreover, the use of the resulting protein preparation has been verified in the mim-tRNAseq pipeline, where it was shown to perform equally to the commercial alternatives Induro and UltraMarathonRT. In addition, we have developed a simple and cost-effective assay for measuring the enzymatic activity of MRT, allowing for batch comparison.

Key features

• Simplified expression and purification of next-generation reverse transcriptase MarathonRT.

• Simple colorimetric assay for measuring the specific activity of MarathonRT, thus enabling the enzyme unit definition.

• Reproducible MarathonRT batches with good storability (at least 12 months) and stable activity.

• Suitable for RNA-sequencing applications and other methods requiring reverse transcription.

## Graphical overview


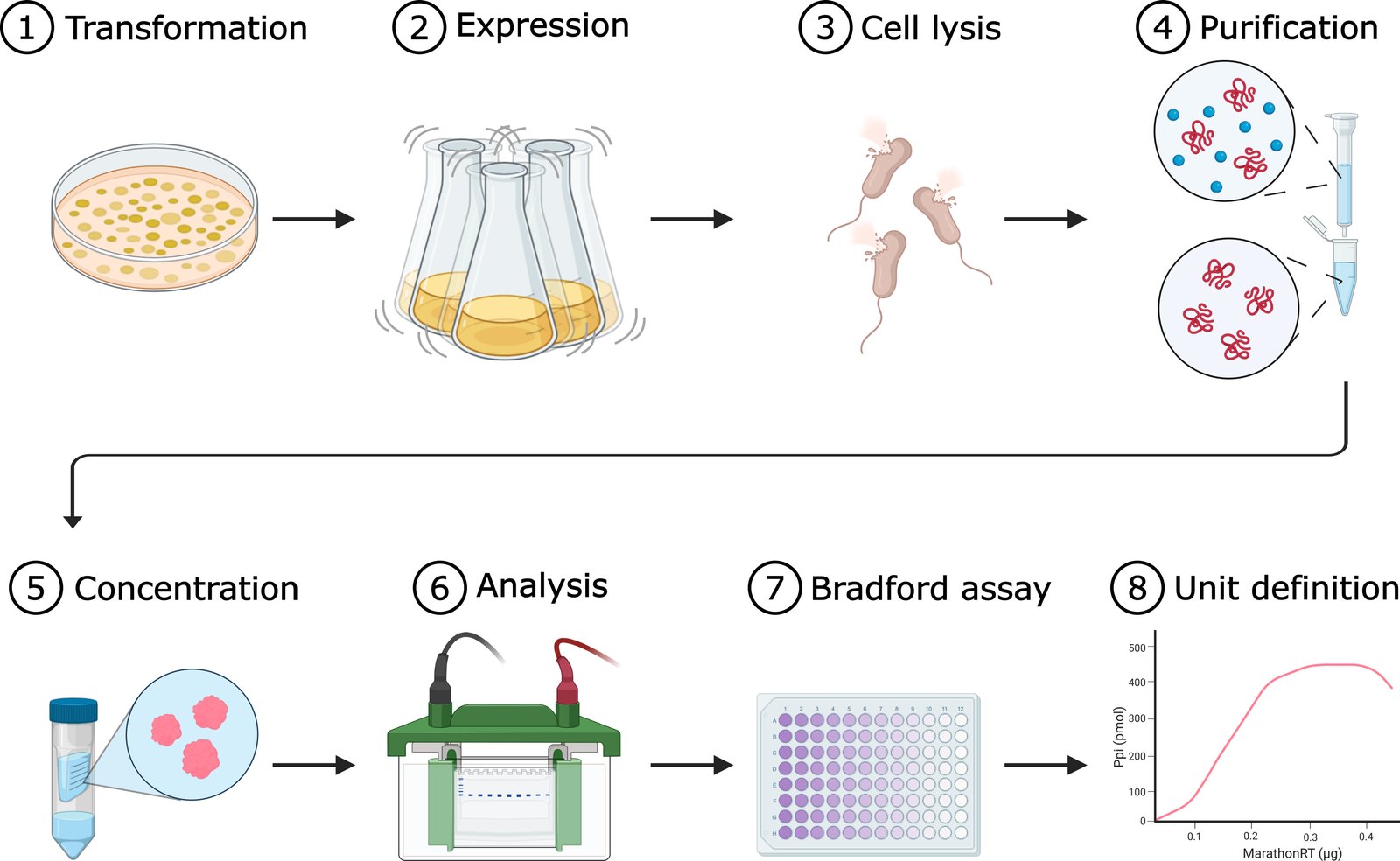


**Simplified workflow for MarathonRT expression and purification (adapted from [1])**

## Background

Reverse transcriptases (RTs) are essential enzymes for converting RNA into cDNA, which have enabled key advances in RNA sequencing, structural probing, and mapping of chemical modifications [2–12]. Over the past decade, successful expression and purification of group II intron-encoded maturases have expanded the RT toolbox with the Thermostable Group II Intron RT (a.k.a. TGIRT) from *Geobacillus stearothermophilus* [7], MarathonRT (MRT) from *Eubacterium rectale* [13], and several engineered, commercial RTs, including UltraMarathonRT (uMRT; RNAConnect) and Induro (New England Biolabs, NEB). UltraMarathonRT is engineered from MarathonRT, but the specific origin of Induro is not publicly available. Unlike conventional retroviral RTs, such as the SuperScript series (Thermo Fisher Scientific), next-generation group II intron-encoded RTs (ngRTs) can efficiently reverse transcribe through strong secondary structures and post-transcriptional modifications (PTMs) [3,7–9,12,14]. Such features are commonplace on transfer RNA (tRNA) molecules [15,16], where they pose significant hurdles for conventional RTs, causing frequent stalling and dissociation from the template. This in turn generates truncated cDNA that hampers the mapping and accurate quantification of tRNAs [7,9,12,14].

Earlier tRNA-seq methods used various strategies to overcome these challenges (reviewed in [17]); these included demethylation of tRNAs prior to reverse transcription to increase the ratio of full-length cDNA, as well as hydrolysis of tRNAs to produce shorter, more manageable tRNA fragments for the RT to process [12,18]. More recently, ngRTs have been successfully used in library preparation without prior treatment of the tRNA, reducing chemical bias and enabling simultaneous readout of sequence and modification signatures [1,3,8]. In parallel, ngRTs have been adopted in direct RNA sequencing (DRS) workflows, where an RT is used to generate an RNA–cDNA duplex that reduces secondary structures [6]. Induro is currently recommended in the SQK-RNA004 protocol by Oxford Nanopore Technologies, and recent work demonstrates similar applicability of uMRT [19].

Although the commercial ngRTs Induro and uMRT have been established as the go-to options for tRNA-seq library preparation workflows [8,9], they remain expensive with high per-reaction costs. Therefore, in-house production of MRT based on published protocols [4,13,14] using a publicly available plasmid presents a budget-conscious solution. Furthermore, it enables a higher degree of user-based customization of the workflow, which furthers the development and implementation of these molecular tools. However, the original MRT expression and purification protocol was developed for resolving the crystal structure of the enzyme, thus aiming for the highest possible purity while sacrificing yield. Here, we present a simplified expression and purification workflow to produce a stable and highly active MRT without contaminating enzymatic activities that is suitable for the RT reaction used in tRNA sequencing library preparation. To this end, we introduced a C-terminal CBD-tag (MRT-CBD), induced expression at lower culture density to reduce degradation by native bacterial proteases, omitted cleavage of N- and C-terminal protein tags (SUMO and CBD), and streamlined the original three-step purification (affinity, ion exchange, and gel filtration) to a single Ni-NTA affinity capture step. For ease of implementation, we also provide a simple and cost-effective colorimetric assay to define MRT and MRT-CBD activity, track storability, and monitor batch-to-batch variation. Under these conditions, we obtained MRT and MRT-CBD preparations of sufficient purity for tRNA-seq RT reactions at an estimated reaction cost of merely 0.0023 EUR and 0.0034 EUR (excluding labor costs) [1], respectively, making this protocol an attractive and widely accessible alternative to commercial ngRTs, where the cost-per-reaction ranges from 11.9 EUR to 19.7 EUR (correct at time of purchase). Moreover, this streamlined MRT production protocol provides the foundation for others to implement and pursue further enhancements to ngRT-related technologies.

While we have demonstrated the successful use of affinity-purified MRT and MRT-CBD in tRNA-seq [1], their suitability for other applications such as single-cell RNA-seq [20] and RT-qPCR [21] should be evaluated separately. An alternative approach could be to use the community-developed MashUp RT (https://pipettejockey.com/), which has been successfully used for reverse transcribing long RNA templates [22,23].

## Materials and reagents

*Note: Equivalent materials and reagents may be used as substitutes.*

**Biological materials**

1. *Escherichia coli* Rosetta 2(DE3)pLysS chemically competent cells, prepared in-house (Merck, Novagen, catalog number: 71403)

2. Plasmid pLJSRSF7-T7-6xHis-SUMO-MarathonRT-CBD (MRT-CBD) (Addgene, catalog number: 253350) originally described in [1] or pET-6xHis-SUMO-MarathonRT (MRT) (Addgene, catalog number: 109029) originally described in [14]

3. Plasmid for RNA template generation (Addgene, catalog number: 101156) originally described in [24]

4. Reverse transcription primer (5′-TCACTGCATACGACGATTCTG-3′) used in the activity assay

**Reagents**

**Antibiotics**

1. Kanamycin sulfate (Thermo Fisher Scientific, Fisher Scientific, Fisher BioReagents, catalog number: BP906-5)

2. Chloramphenicol (Thermo Fisher Scientific, Fisher Scientific, Fisher BioReagents, catalog number: BP904-100)

**Inducers and reducing agents**

3. Isopropyl-B-D-thiogalactopyranoside (IPTG) (Thermo Fisher Scientific, Fisher Scientific, Thermo Scientific, catalog number: R0392)

4. Dithiothreitol (DTT) (Thermo Fisher Scientific, Fisher Scientific, Fisher BioReagents, catalog number: BP172-25)

5. β-Mercaptoethanol (Thermo Fisher Scientific, Fisher Scientific, Thermo Scientific Acros, catalog number: 125472500)

**Buffers, acids, and bases**

6. HEPES [4-(2-hydroxyethyl)-1-piperazineethanesulfonic acid] (Thermo Fisher Scientific, Fisher Scientific, Fisher BioReagents, catalog number: BP310-1)

7. Tris base [Tris(hydroxymethyl)aminomethane] (Thermo Fisher Scientific, Fisher Scientific, Fisher BioReagents, catalog number: BP152-1)

8. Hydrochloric acid (HCl) for pH adjustment (any)

9. Sulfuric acid (Thermo Fisher Scientific, Fisher Scientific, Fisher Chemicals, catalog number: S/9240/PB15)

10. Sodium hydroxide (NaOH) (Thermo Fisher Scientific, Fisher Scientific, Fisher Chemical, catalog number: S/4920/60)

11. Potassium hydroxide (KOH) (Thermo Fisher Scientific, Fisher Scientific, Fisher Chemical, catalog number: P/5560/60)

**Salts**

12. Sodium chloride (NaCl) (Thermo Fisher Scientific, Fisher Scientific, Fisher Chemical, catalog number: S/3120/63)

13. Potassium chloride (KCl) (Thermo Fisher Scientific, Fisher Scientific, Fisher Chemical, catalog number: P/4240/60)

14. Magnesium chloride (MgCl_2_) (Thermo Fisher Scientific, Fisher Scientific, Fisher BioReagents, catalog number: 10386743)

15. Sodium phosphate monobasic anhydrous (NaH_2_PO_4_) (Thermo Fisher Scientific, Fisher Scientific, Fisher BioReagents, catalog number: BP329500)

16. Sodium phosphate dibasic anhydrous (Na_2_HPO_4_) (Thermo Fisher Scientific, Fisher Scientific, Fisher BioReagents, catalog number: BP332-500)

17. Potassium phosphate, monobasic (KH_2_PO_4_) (Thermo Fisher Scientific, Fisher Scientific, Thermo Scientific Acros, catalog number: 205925000)

**Amino acids/protein components**

18. BSA (albumin, bovine) (Avantor, VWR Life Science, catalog number: 0332-100G)

**Organic compounds and solvents**

19. Glycerol, ≥99%, analytical reagent grade (Thermo Fisher Scientific, Fisher Scientific, Fisher Chemical, catalog number: G/0650/08)

20. Ethanol (EtOH), AA grade (Anora Group Oyj, Anora Industrial, Etax Aa, catalog number: 1025874)

21. Tween 20 (Thermo Fisher Scientific, Fisher Scientific, Fisher BioReagents, catalog number: BP337-500)

22. Orthophosphoric acid, >85%, extra pure, SLR 500 mL (Thermo Fisher Scientific, Fisher Scientific, Fisher Chemical, catalog number: 10122010)

23. Ethylene glycol diacetate (EGDA) (Thermo Fisher Scientific, Fisher Scientific, Thermo Scientific Acros, catalog number: 10022400)

**Chaotropic agents and denaturants**

24. Guanidine thiocyanate (Thermo Fisher Scientific, Fisher Scientific, Thermo Scientific Chemicals, catalog number: 411112500)

**Imidazole derivatives**

25. Imidazole (Thermo Fisher Scientific, Fisher Scientific, Thermo Scientific Acros, catalog number: 122025000)

**Colorimetric reagents**

26. Coomassie Blue G250 (Thermo Fisher Scientific, Fisher Scientific, Fisher BioReagents, catalog number: C/P541/46)

27. Malachite green (oxalate) (Thermo Fisher Scientific, Fisher Scientific, Fisher Chemical, catalog number: M/1245/46)

28. Ammonium molybdate (IV) tetrahydrate (Thermo Fisher Scientific, Fisher Scientific, Thermo Scientific Acros, catalog number: 205851000)

**Media components**

29. Tryptone (Neogen, catalog number: NCM0120A)

30. Yeast extract (Neogen, catalog number: NCM0218)

31. Agar (Neogen, catalog number: NCM0238A)

**Enzymes**

32. *Eco*RI (Thermo Fisher Scientific, Fisher Scientific, Thermo Scientific, catalog number: FD0274)

33. FastAP thermosensitive alkaline phosphatase (Thermo Fisher Scientific, Fisher Scientific, Thermo Scientific, catalog number: EF0654)

34. T7 polymerase (20 U/μL) (Thermo Fisher Scientific, Fisher Scientific, Thermo Scientific, catalog number: EP0111)

35. RQ1 RNase-free DNase (Promega, catalog number: M6101)

36. RNasin Plus ribonuclease inhibitor 10,000 U (Promega, catalog number: N2615)

37. Pyrophosphatase, inorganic (0.1 U/μL) (Thermo Fisher Scientific, Fisher Scientific, Thermo Scientific, catalog number: EF0221)

**Nucleotides**

38. dNTP set, 100 mM solutions (Thermo Fisher Scientific, Fisher Scientific, Thermo Scientific, catalog number: R0181)

39. NTP set, 100 mM solutions (Thermo Fisher Scientific, Fisher Scientific, Thermo Scientific, catalog number: R0481)

**Gel electrophoresis**

40. SDS-PAGE 10% gels, Tris-Glycine buffering system for proteins (any)

41. Urea-PAA 5% gel, TBE buffering system for RNA (any)

42. Agarose gel 1% gel, TBE or TAE buffering system for DNA (any)

43. Midori Green Advance (Nippon Genetics, catalog number: MG04)

44. SYBR Gold nucleic acid gel stain (10,000× concentrate in DMSO) (Thermo Fisher Scientific, Fisher Scientific, Invitrogen, catalog number: S11494)

45. InstantBlue Coomassie protein stain (ISB1L) (Abcam, catalog number: ab119211)

46. PageRuler Plus Prestained protein ladder, 10–250 kDa (Thermo Fisher Scientific, Fisher Scientific, Thermo Scientific, catalog number: 26619)

47. ssRNA ladder (New England Biolabs, catalog number: N0362S)

48. GeneRuler 1 kb DNA ladder (Thermo Fisher Scientific, Fisher Scientific, Thermo Scientific, catalog number: SM0311)

**Specialized materials and kits:**

49. Liquid nitrogen

50. NucleoSpin Gel and PCR Clean-up XS (Macherey-Nagel, catalog number: 740611.250)

51. NucleoSpin RNA Columns for RNA purification (Macherey-Nagel, catalog number: 740955.50S)

52. Qubit RNA Broad Range (BR) Assay kit (Thermo Fisher Scientific, Fisher Scientific, Invitrogen, catalog number: Q10211)

**Solutions**

1. Kanamycin, 50 mg/mL (see Recipes)

2. Chloramphenicol, 35 mg/mL (see Recipes)

3. IPTG, 1 M (see Recipes)

4. Glycerol, 80% (see Recipes)

5. Glycerol, 90% (see Recipes)

6. Imidazole, 1 M (see Recipes)

7. NaOH, 5 M (see Recipes)

8. KOH, 5 M (see Recipes)

9. NaCl, 5 M (see Recipes)

10. KCl, 3 M (see Recipes)

11. MgCl_2_, 4 M (see Recipes)

12. DTT, 1 M (see Recipes)

13. K-HEPES pH 7.5, 1 M (see Recipes)

14. Na-HEPES pH 7.5, 1 M (see Recipes)

15. Tris-HCl pH 8.3, 1 M (see Recipes)

16. Tris-HCl pH 7.5, 1 M (see Recipes)

17. Tris-HCl pH 7.0, 1 M (see Recipes)

18. BSA 10 μg/μL (see Recipes)

19. BSA 0.1 μg/μL (see Recipes)

20. Guanidine thiocyanate, 5 M (see Recipes)

21. Solutions for measuring MRT activity

a. Inorganic phosphate solution (Pi), 100 mM (see Recipes)

b. Malachite green reagent, 0.12% in 3 M H_2_SO_4_ (see Recipes)

c. Ammonium molybdate, 7.5% (w/v) (see Recipes)

d. Tween 20, 10% (v/v) (see Recipes)

22. Buffers for MRT expression

a. LB Miller broth (see Recipes)

b. LB Miller agar (see Recipes)

c. PBS, 10× (see Recipes)

d. PBS, 1× (see Recipes)

23. Buffers for MRT purification

a. Buffer A (see Recipes)

b. Buffer B (see Recipes)

c. Buffer C (see Recipes)

d. Buffer D (see Recipes)

e. Buffer G (-glycerol) (see Recipes)

f. Buffer G (+glycerol)/storage buffer (see Recipes)

24. Buffers for Bradford Assay

a. Bradford reagent (see Recipes)

25. Buffers for RNA template purification [25]

a. Lysis/binding buffer (LBB) (see Recipes)

b. Chaotropic wash (CW) (see Recipes)

c. Ethanol wash (EW) (see Recipes)

26. Buffers for MarathonRT unit definition

a. MRT 2× reaction buffer (see Recipes)

b. Pi dilutions (for standard curve) (see Recipes)

c. Malachite green detection solution (DS) (see Recipes)

**Recipes**

**1. Kanamycin, 50 mg/mL (25 mL)**

Weigh 1.25 g of kanamycin into a 50 mL conical centrifuge tube. Add nuclease-free water up to 20 mL and dissolve kanamycin using an end-over-end mixer. Adjust the volume to 25 mL and filter through a disposable 0.2 μm 150 mL filter into a sterile 100 mL glass bottle. Aliquot 1 mL into 1.5 mL microfuge tubes. Store at -20 °C indefinitely.

**2. Chloramphenicol, 35 mg/mL (25 mL)**

Weigh 0.88 g of chloramphenicol into a 50 mL conical centrifuge tube. Add nuclease-free water up to 20 mL and dissolve chloramphenicol using an end-over-end mixer. Adjust the volume to 25 mL and filter through a disposable 0.2 μm 150 mL filter into a sterile 100 mL glass bottle. Aliquot 1 mL into 1.5 mL microfuge tubes. Store at -20 °C indefinitely.

**3. IPTG, 1 M (20 mL)**

Weigh 4.77 g of IPTG into a 50 mL conical centrifuge tube. Add nuclease-free water up to 15 mL and dissolve IPTG using an end-over-end mixer. Adjust the volume to 20 mL in a volumetric bottle or cylinder and filter through a disposable 0.2 μm 150 mL filter into a sterile 100 mL glass bottle. Aliquot 1 mL into 1.5 mL microfuge tubes. Store at -20 °C for up to 2 years.

**4. Glycerol, 80% (80 mL)**

Weigh 64 g of glycerol into a 100 mL glass bottle. Add 16 g of nuclease-free water. Mix over the cap 5–10×. Autoclave in liquid cycle. Store at room temperature indefinitely.

**5. Glycerol, 90% (80 mL)**

Weigh 72 g of glycerol into a 100 mL glass bottle. Add 8 g of nuclease-free water. Mix over the cap 5–10×. Autoclave in liquid cycle. Store at room temperature indefinitely.

**6. Imidazole, 1 M (100 mL)**

Weigh 6.81 g of imidazole into a 100 mL beaker. Add nuclease-free water up to ~80 mL and dissolve imidazole using a magnetic stirrer. Adjust the volume to 100 mL in a volumetric bottle or cylinder and filter through a disposable 0.2 μm 150 mL filter into a sterile 100 mL glass bottle. Store at 4 °C protected from light for up to 2 years.

**7. NaOH, 5 M (100 mL)**

Weigh 20 g of NaOH pellets. Add NaOH slowly to water (do not add water to pellets) and stir in a 250–500 mL beaker with cooling if needed. Start with ~80 mL of nuclease-free water, dissolve, then adjust to 100 mL in a volumetric bottle or cylinder. Aliquot into 50 mL conical centrifuge tubes. Store at room temperature indefinitely.

**8. KOH, 5 M (100 mL)**

Weigh 28.06 g of KOH pellets. Add KOH slowly to water (do not add water to pellets) and stir in a 250–500 mL beaker with cooling if needed. Start with ~80 mL of nuclease-free water, dissolve, then adjust to 100 mL in a volumetric bottle or cylinder. Aliquot into 50 mL conical centrifuge tubes. Store at room temperature indefinitely.

**9. NaCl, 5 M (100 mL)**

Weigh 29.22 g of NaCl into a 250 mL beaker. Add nuclease-free water up to 80 mL and dissolve NaCl using a magnetic stirrer. Adjust the volume to 100 mL in a volumetric bottle or cylinder and filter through a disposable 0.2 μm 150 mL filter into a sterile 100 mL glass bottle. Store at room temperature indefinitely.

**10. KCl, 3 M (100 mL)**

Weigh 22.36 g of KCl into a 250 mL beaker. Add nuclease-free water up to 80 mL and dissolve KCl using a magnetic stirrer. Adjust the volume to 100 mL in a volumetric bottle or cylinder and filter through a disposable 0.2 μm 150 mL filter into a sterile 100 mL glass bottle. Store at room temperature indefinitely.

**11. MgCl_2_, 4 M (100 mL)**

Weigh 50.34 g of MgCl_2_ into a 250 mL beaker. Add ~80 mL of nuclease-free water. Use a magnetic mixer to dissolve MgCl_2_. Set the final volume to 100 mL with nuclease-free water using a volumetric bottle or a cylinder and filter through a disposable 0.2 μm 150 mL filter into a sterile 100 mL glass bottle. Store at room temperature indefinitely.

**12. DTT, 1 M (25 mL)**

Weigh 3.86 g of DTT into a 50 mL conical centrifuge tube. Add nuclease-free water up to 20 mL and dissolve DTT using an end-over-end mixer. Adjust the volume to 25 mL and filter through a disposable 0.2 μm 150 mL filter into a sterile 100 mL glass bottle. Aliquot 1 mL into 1.5 mL microfuge tubes. Store at -20 °C up to 2 years.

**13. K-HEPES pH 7.5, 1 M (100 mL)**

Weigh 23.83 g of HEPES into a 250 mL beaker. Add ~80 mL of nuclease-free water. Use a magnetic mixer to dissolve HEPES. Once dissolved, adjust the pH with 5 M KOH solution. Set the final volume to 100 mL with nuclease-free water using a volumetric bottle or a cylinder and filter through a disposable 0.2 μm 150 mL filter into a sterile 100 mL glass bottle. Aliquot 10 mL into 15 mL centrifuge tubes and store at -20 °C for up to 2 years.

**Critical:** HEPES buffers are light- and temperature-sensitive. HEPES solution is stable at 4 °C for up to a month when protected from light.

**14. Na-HEPES pH 7.5, 1 M (100 mL)**

Weigh 23.83 g of HEPES into a 250 mL beaker. Add ~80 mL of nuclease-free water. Use a magnetic mixer to dissolve HEPES. Once dissolved, adjust the pH with 5 M NaOH solution. Set the final volume to 100 mL with nuclease-free water using a volumetric bottle or a cylinder and filter through a disposable 0.2 μm 150 mL filter into a sterile 100 mL glass bottle. Aliquot 10 mL into 15 mL centrifuge tubes and store at -20 °C for up to 2 years.

**Critical:** HEPES buffers are light- and temperature-sensitive. HEPES solution is stable at 4 °C for up to a month when protected from light.

**15. Tris-HCl pH 8.3, 1 M (100 mL)**

Weigh 12.11 g of Tris base into a 250 mL beaker. Add ~80 mL of nuclease-free water. Use a magnetic mixer to dissolve Tris base. Once dissolved, adjust the pH with HCl solution. Set the final volume to 100 mL with nuclease-free water using a volumetric bottle or a cylinder and filter through a disposable 0.2 μm 150 mL filter into a sterile 100 mL glass bottle. Store at room temperature for up to 1 year.

**16. Tris-HCl pH 7.5, 1 M (100 mL)**

Weigh 12.11 g of Tris base into a 250 mL beaker. Add ~80 mL of nuclease-free water. Use a magnetic mixer to dissolve Tris base. Once dissolved, adjust the pH with HCl solution. Set the final volume to 100 mL with nuclease-free water using a volumetric bottle or a cylinder and filter through a disposable 0.2 μm 150 mL filter into a sterile 100 mL glass bottle. Store at room temperature for up to 1 year.

**17. Tris-HCl pH 7.0, 1 M (100 mL)**

Weigh 12.11 g of Tris base into a 250 mL beaker. Add ~80 mL of nuclease-free water. Use a magnetic mixer to dissolve Tris base. Once dissolved, adjust the pH with HCl solution. Set the final volume to 100 mL with nuclease-free water using a volumetric bottle or a cylinder and filter through a disposable 0.2 μm 150 mL filter into a sterile 100 mL glass bottle. Store at room temperature for up to 1 year.

**18. BSA 10 μg/L (10 mL)**

Weigh 0.1 g of BSA into a 50 mL tube. Add ~8 mL of nuclease-free water. Use an end-over-end mixer to dissolve BSA. Avoid foam. Set the final volume to 10 mL with nuclease-free water using a volumetric bottle or a cylinder and aliquot into microfuge tubes. Store at -20 °C for years. Avoid freeze-thaws.

**19. BSA 0.1 μg/μL (1 mL)**

Pipette 10 μL of 10 μg/μL BSA into 990 μL of nuclease-free water. Mix by pipetting up and down. Use immediately.

**20. Guanidine thiocyanate, 5 M (10 mL)**

Weigh 5.91 g of guanidine thiocyanate into a 50 mL tube. Add ~8 mL of nuclease-free water. Use a magnetic mixer with heating to dissolve it. Set the final volume to 10 mL with nuclease-free water using a volumetric bottle or a cylinder and transfer into a 15 mL tube. Store at room temperature protected from light for years.

**21. Solutions for measuring MRT activity**

**Critical:** Use only sterile plasticware for preparation of these solutions, except for malachite green reagent. Nuclease-free water must be taken directly from the water purification device into plasticware. Glassware might contain trace amounts of phosphate, which will interfere with the assay. Do not sterilize these solutions.

**a. Inorganic phosphate solution (P_i_), 100 mM (10 mL)**

Dissolve 0.12 g of NaH_2_PO_4_ (119.98 g/mol) in 7–8 mL of nuclease-free water in a 15 mL tube. Adjust the final volume to 10 mL with nuclease-free water in the same tube. Do not filter-sterilize. Store at 4 °C for 1–2 months.

**b. Malachite green reagent, 0.12% in 3 M H_2_SO_4 _(180 mL)**

Prepare 3 M sulfuric acid by diluting 30 mL of concentrated sulfuric acid in 150 mL of nuclease-free water in a well-rinsed glass bottle. Add 0.22 g of malachite green and mix well. This yields a 0.12% malachite green solution in 3 M (6 N) sulfuric acid. Do not filter-sterilize. The solution is stable at room temperature for up to 1 year. Always store the solution in glassware!

**c. Ammonium molybdate, 7.5% (w/v) (10 mL)**

Dissolve 0.75 g of ammonium molybdate tetrahydrate in 5 mL of nuclease-free water in a 15 mL tube. Once dissolved, adjust the volume to 10 mL with nuclease-free water in the same tube. Do not filter-sterilize. The solution is stable indefinitely if stored in a plastic bottle. However, the solution may occasionally precipitate. If this happens, prepare a new solution.

**d. Tween 20, 10% (v/v) (10 mL)**

Dilute 1 mL of concentrated Tween 20 in 5–6 mL of nuclease-free water in a 50 mL tube. Mix properly on an end-over-end rotator. Adjust the volume to 10 mL with nuclease-free water. Store at room temperature. Do not filter-sterilize.

**22. Buffers for MRT expression**

**a. LB Miller broth (1 L)**

**Table:** 

Reagent	Final concentration	Quantity or volume
Tryptone	10 g/L	10 g
Yeast extract	5 g/L	5 g
NaCl	10 g/L	10 g
Reverse osmosis (RO) water	N/A	Up to 1 L
Total volume		1 L

Weigh all components into a 1 L beaker. Add RO water up to 900 mL and dissolve using a magnetic stirrer. Adjust the volume to 1 L in a volumetric cylinder or bottle. Dispense 500 mL into 1 L glass bottles and sterilize by autoclaving at 121 °C for 15 min. Store at room temperature for years.

**b. LB Miller agar (250 mL)**

**Table:** 

Reagent	Final concentration	Quantity or volume
Tryptone	10 g/L	2.5 g
Yeast extract	5 g/L	1.25 g
NaCl	10 g/L	2.5 g
Agar	15 g/L	3.75 g
Nuclease-free water	N/A	Up to 250 mL
Total volume		250 mL

Weigh all components into a 500 mL beaker. Add nuclease-free water up to 200 mL and dissolve using a magnetic stirrer. Adjust the volume to 250 L in a volumetric cylinder or bottle. Dispense 250 mL into 100 mL glass bottles and sterilize by autoclaving at 121 °C for 15 min. Store at room temperature for years.

**c. PBS, 10× (100 mL)**

**Table:** 

Reagent	Final concentration	Quantity or volume
NaCl	1.37 M	8 g
KCl	27 mM	0.2 g
Na_2_HPO_4_	100 mM	1.42 g
KH_2_PO_4_	18 mM	0.24 g
Nuclease-free water	N/A	Up to 100 mL
Total volume		100 mL

Weigh all components into a 250 mL beaker. Add nuclease-free water up to 90 mL and dissolve using a magnetic stirrer. Adjust the volume to 100 mL in a volumetric bottle or cylinder and filter through a disposable 0.2 μm 150 mL filter into a sterile 100 mL glass bottle. Store at room temperature indefinitely.

**d. PBS, 1× (100 mL)**

Pipette 10 mL of 10× PBS stock into a volumetric bottle or cylinder. Bring the volume to 100 mL and transfer to a 100 mL glass bottle. Mix over the cap. Store at room temperature indefinitely.

**23. Buffers for MRT purification**

The buffer nomenclature is the same as in the original publication [13].

We recommend that all buffers be prepared a day before the purification of the protein and always stored at 4 °C. Sterilize all solutions before making the buffers. We recommend filter-sterilizing all self-prepared solutions made of powders and only autoclaving glycerol. Add β-Mercaptoethanol and DTT to the buffers just before use. These components are unstable and will render the buffers not usable the next day.

**a. Buffer A**

**Table:** 

Reagent	Final concentration	Quantity or volume
Na-HEPES pH 7.5 (1 M)	25 mM	1.5 mL
NaCl, (5 M)	1 M	12 mL
Glycerol (80%)	10%	7.5 mL
β-Mercaptoethanol (14.3 M)	2 mM	8.4 μL
Nuclease-free water	N/A	39 mL
Total volume		60 mL

**b. Buffer B**

**Table:** 

Reagent	Final concentration	Quantity or volume
Na-HEPES pH 7.5 (1 M)	25 mM	0.75 mL
NaCl (5 M)	500 mM	3 mL
Imidazole (1 M)	20 mM	0.6 mL
Glycerol (80%)	10%	3.75 mL
β-Mercaptoethanol (14.3 M)	2 mM	4.2 μL
Nuclease-free water	N/A	21.9 mL
Total volume		30 mL

**c. Buffer C**

**Table:** 

Reagent	Final concentration	Quantity or volume
Na-HEPES pH 7.5 (1 M)	25 mM	0.75 mL
NaCl (5 M)	500 mM	3 mL
Imidazole (1 M)	30 mM	0.9 mL
Glycerol (80%)	10%	3.75 mL
β-Mercaptoethanol (14.3 M)	2 mM	4.2 μL
Nuclease-free water	N/A	21.6 mL
Total volume		30 mL

**d. Buffer D**

**Table:** 

Reagent	Final concentration	Quantity or volume
Na-HEPES pH 7.5 (1 M)	25 mM	0.5 mL
NaCl (5 M)	300 mM	1.2 mL
Imidazole (1 M)	300 mM	6 mL
Glycerol (80%)	10%	2.5 mL
β-Mercaptoethanol (14.3 M)	2 mM	2.8 μL
Nuclease-free water	N/A	9.8 mL
Total volume		20 mL

**e. Buffer G (-glycerol)**

**Table:** 

Reagent	Final concentration	Quantity or volume
K-HEPES pH 7.5 (1 M)	56.25 mM	1.69 mL
KCl (3 M)	675 mM	6.75 mL
DTT (1 M)	2.25 mM	67.5 μL
Nuclease-free water	N/A	21.5 mL
Glycerol (90%)	N/A	N/A
Total volume		30 mL

**f. Buffer G (+glycerol)/storage buffer**

**Table:** 

Reagent	Final concentration	Quantity or volume
K-HEPES pH 7.5 (1 M)	25 mM	250 μL
KCl (3 M)	300 mM	1 mL
DTT (1 M)	1 mM	10 μL
Glycerol (90%)	50%	6.25 mL
Nuclease-free water	N/A	2.49 mL
Total volume		10 mL

We recommend that the buffer be prepared fresh, aliquoted into 1.5 mL microfuge tubes, and stored at -20 °C. We have successfully used the buffer stored for 2 years.

**24. Buffers for Bradford assay**

**a. Bradford reagent**

**Table:** 

Reagent	Final concentration	Quantity or volume
Coomassie Brilliant Blue G250	N/A	0.1 g
EtOH (96%–100%)	10%	50 mL
Orthophosphoric acid	20%	100 mL
Nuclease-free water	N/A	350 mL
Total volume		500 mL

Weigh 0.1 g of Coomassie Brilliant Blue G250. Use a magnetic mixer to dissolve it in 50 mL of ethanol in a 1 L beaker for 1 h. Add 100 mL of orthophosphoric acid and stir. Add 350 mL of nuclease-free water and stir. Aliquot into 50 mL glass bottles. Store at 4 °C protected from light.

**25. Buffers for RNA template purification [25]**

**a. Lysis/binding buffer (LBB)**

**Table:** 

Reagent	Final concentration	Quantity or volume
Guanidine thiocyanate	5 M	5.91 g
Tris-HCl pH 7.0 (1 M)	50 mM	0.5 mL
Nuclease-free water	N/A	Up to 10 mL
Total volume		10 mL

Weigh 5.91 g of guanidine thiocyanate into a 50 mL beaker. Add ~8 mL of nuclease-free water and 0.5 mL of Tris-HCl pH 7.0. Use a magnetic mixer with heating to dissolve guanidine thiocyanate. Once dissolved, adjust the final volume to 10 mL with nuclease-free water using a volumetric bottle or a cylinder. Transfer to a 15 mL tube and store at room temperature for up to 6 months.

**b. Chaotropic wash (CW)**

**Table:** 

Reagent	Final concentration	Quantity or volume
Tris-HCl pH 7.0 (1 M)	20 mM	200 μL
Guanidine thiocyanate (5 M)	1.6 M	3.2 mL
EtOH (99.6%)	66%	6.6 mL
Total volume		10 mL

**c. Ethanol wash (EW)**

**Table:** 

Reagent	Final concentration	Quantity or volume
Tris-HCl pH 7.5 (1 M)	10 mM	100 μL
EtOH (99.6%)	80%	8 mL
NaCl (5 M)	20 mM	40 μL
Nuclease-free water	N/A	1.86 mL
Total volume		10 mL

Pipette the components in the following order: water, Tris-HCl, NaCl, and EtOH to avoid salt precipitation in ethanol.

**26. Buffers for MarathonRT unit definition**

**a. MRT 2× reaction buffer**

**Table:** 

Reagent	Final concentration	Quantity or volume	In 1× reaction
Tris-HCl pH 8.3 (1 M)	100 mM	1 mL	50 mM
KCl (3 M)	400 mM	1.33 mL	200 mM
MgCl_2 _(4 M)	4 mM	10 μL	2 mM
DTT (1 M)	10 mM	100 μL	5 mM
Glycerol (80%)	40%	5 mL	20%
Nuclease-free water	N/A	2.56 mL	N/A
Total volume		10 mL	

We recommend that the buffer be prepared fresh, aliquoted into 1.5 mL microfuge tubes, and stored at -20 °C. We have successfully used the buffer stored for 2 years.

*Note: This original MRT reaction buffer with 2 mM MgCl_2_ was used for establishing the assay. An optimized MRT reaction buffer with 4 mM MgCl_2_ was subsequently released (uMRT RT kit protocol by RNAConnect, previously available from Kerafast). However, we retained the original buffer composition in order to compare the activity of all protein batches. A new user may find it beneficial to use the optimized buffer with a higher MgCl_2 _concentration.*

**b. P_i_ dilutions (for standard curve)**

**Table:** 

Plate row	P_i_ 50 μm (μL)	Ultrapure water (μL)	Amount in detection reaction
A	0 μL	80 μL	0 pmol (0 μm)
B	6 μL	74 μL	300 pmol (3 μm)
C	12 μL	68 μL	600 pmol (6 μm)
D	24 μL	56 μL	1,200 pmol (12 μm)
E	40 μL	40 μL	2,000 pmol (20 μm)
F	50 μL	30 μL	2,500 pmol (25 μm)
G	64 μL	16 μL	3,200 pmol (32 μm)
H	80 μL	0 μL	4,000 pmol (40 μm)

**Critical:** P_i_ dilutions must be prepared just before use!

Prepare the P_i_ dilutions for the standard curve from the P_i_ stock (Recipe 21a). First, prepare 1 mM P_i_ by pipetting 990 μL of nuclease-free water into a 1.5 mL microfuge tube and adding 10 μL of 100 mM P_i_. Mix by vortex and pulse-spin. Second, prepare 50 μM P_i_ by pipetting 950 μL of nuclease-free water into a 1.5 mL microfuge tube and adding 50 μL of 1 mM P_i_ from the previous step. Mix by vortex and pulse-spin. Next, prepare standard curve dilutions of the freshly prepared 50 μM P_i_ directly to the microplate. First, pipette ultrapure water; then, add P_i_.

**c. Malachite green detection solution (DS)**

**Table:** 

Component	For 1 reaction	For 30 reactions	For 100 reactions
Malachite green reagent	15.73 μL	472 μL	1,574 μL
Ammonium molybdate (7.5%)	3.93 μL	118 μL	394 μL
Tween (10%)	0.33 μL	10 μL	32 μL

**Critical:** Malachite green DS must be prepared just before use!

Prepare the malachite green DS by mixing malachite green reagent (Recipe 21b) with 7.5% ammonium molybdate (Recipe 21c) and 10% Tween (Recipe 21d) into a 1.5 or 2 mL microfuge tube, as shown in the table above.

**Critical:** Always mix in the following order: malachite green, ammonium molybdate, and Tween-20. Otherwise, the solution will precipitate.

**Laboratory supplies**

1. MaxyClear Snaplock Microtubes, 1.5 mL (Thermo Fisher Scientific, Fisher Scientific, Axygen, catalog number: MCT150C) or equivalent

2. Screw cap tube, 15 mL, (L × Ø): 120 × 17 mm, PP, with print (Sarstedt, catalog number: 62.554.002) or equivalent

3. Screw cap tube, 50 mL, (L × Ø): 114 × 28 mm, PP, with print (Sarstedt, catalog number: 62.547.255) or equivalent

4. PCR tubes with 0.5 mL flat cap (Thermo Fisher Scientific, Fisher Scientific, Axygen, catalog number: PCR05C)

5. 0.2 mL PCR 8-well strips with single flat caps (Nippon Genetics, catalog number: FG-088WF)

6. 10 μL pipette tips nuclease-free (any)

7. 200 μL pipette tips nuclease-free (any)

8. 1 mL pipette tips nuclease-free (any)

9. Serological pipette, with tip, plugged, 10 mL, sterile, non-pyrogenic/endotoxin-free, non-cytotoxic, 1 piece(s)/blister (Sarstedt, catalog number: 86.1254.001) or equivalent

10. Serological pipette, with tip, plugged, 25 mL, sterile, non-pyrogenic/endotoxin-free, non-cytotoxic, 1 piece(s)/blister (Sarstedt, catalog number: 86.1685.001) or equivalent

11. Petri dishes (any)

12. Glass beads/cell spreader (any)

13. 250 mL Erlenmeyer flask (any)

14. 2 L Erlenmeyer flask (any)

15. Glass bottles for buffers and media (any)

16. Volumetric flasks for buffer preparation (any)

17. Glass beakers (any)

18. Sterile inoculation loops (any)

19. Disposable cuvettes PS semi-micro (Avantor, VWR, catalog number: 634-0676)

20. Fiberlite 1000 mL bottles (Thermo Fisher Scientific, Fisher Scientific, Thermo Scientific, catalog number: 010-1491) or Nalgene PPCO centrifuge bottles 500 mL (Thermo Fisher Scientific, Fisher Scientific, Thermo Scientific, catalog number: 3120-0500) or equivalent

21. Nalgene Oak Ridge Polysulfone centrifuge tubes w/sealing caps, 50 mL (Thermo Fisher Scientific, Fisher Scientific, Thermo Scientific, catalog number: 31370050) or equivalent

22. Pierce centrifuge columns, 10 mL (Thermo Fisher Scientific, Fisher Scientific, Thermo Scientific, catalog number: 89898)

23. Disposable PES bottle top filters 150 mL (Thermo Fisher Scientific, Fisher Scientific, Fisherbrand, catalog number: 15953307)

24. Disposable PES bottle top filters, 500 mL (Thermo Fisher Scientific, Fisher Scientific, Fisherbrand, catalog number: 15973307)

25. Amicon Ultra centrifugal filter, 50 kDa MWCO (Merck, catalog number: UFC905024)

26. HisPur Ni-NTA resin (Thermo Fisher Scientific, Fisher Scientific, Thermo Scientific, catalog number: 88221)

27. Nunc 96-well microplate (Thermo Fisher Scientific, Fisher Scientific, Thermo Scientific, catalog number: 260836)

28. (Optional) TapeStation Screentapes for RNA (Agilent)

29. (Optional) Multichannel reagent reservoir, any

## Equipment

1. 4 °C fridge

2. Cold room

3. -70 °C freezer

4. -20 °C freezer

5. Ice box

6. Direct 8 Milli-Q Direct Water Purification System with filter Biopak Polisher (Merck, catalog number: CDUFBI001)

7. QB Series Dry Block Heating Systems (Grant, model: QBD2) or equivalent

8. XT^96^ Thermal Cycler XT96 Gradient (Avantor, VWR, catalog number: 732-3428) or equivalent

9. ThermoMixer C (Eppendorf, catalog number: 5382000015) or equivalent

10. Incubator shaker New Brunswick^TM^ Excella E25 Shaker (Eppendorf) or equivalent temperature-controlled shaker that can fit a 2 L flask

11. Microbiological incubator (any)

12. NanoDrop 2000c Spectrophotometer (Thermo Fisher Scientific, Thermo Scientific, catalog number: ND-2000C) or equivalent

13. Superspeed Centrifuge Sorvall LYNX 4000 with rotors Fiberlite F20-12 × 50 LEX, Fiberlite F12-6 × 500 LEX or Fiberlite F10-4 × 1000 LEX (Thermo Fisher Scientific, Thermo Scientific, catalog number: 75006581) or equivalent

14. Centrifuge 5427 R with rotor FA-45-24-11 (Eppendorf, catalog number: 5429000010) or equivalent temperature-controlled centrifuge that fits 1.5/2 mL microfuge tubes

15. Centrifuge 5810 R with rotors fixed-angle FA-45-6-30 and optional: Swing-bucket rotor A-4-62 (Eppendorf, catalog number: 5811000015) or equivalent

16. Balance AB104-S/PH (Mettler Toledo, catalog number: 11135020) or equivalent

17. Top-loading balance (Sartorius, model: CP2202S) or equivalent

18. Stripettor Ultra Pipet Controller (Corning, catalog number: 4099) or equivalent

19. Electrophoresis Power Supply (CBS Scientific, model: EPS-600) or equivalent

20. Agarose gel electrophoresis equipment (any)

21. Open Air Rocker (Thermo Fisher Scientific, Fisher Scientific, Fisherbrand, catalog number: 88861026) or equivalent

22. ChemiDoc MP Imaging System (Bio-Rad, catalog number: 12003154) or equivalent

23. Mini-PROTEAN Tetra Vertical Electrophoresis Cell (Bio-Rad, catalog number: 1658004) or equivalent

24. Dual Adjustable Vertical System for polyacrylamide gel electrophoresis (CBS Scientific, catalog number: DASG-250) or equivalent

25. (Optional) Automatic gel electrophoresis equipment for DNA and RNA 4150 TapeStation System (Agilent, catalog number: G2992AA)

26. Research Plus 0.5–10 μL pipette (Eppendorf, catalog number: EP3123000020) or equivalent

27. Research Plus 10–100 μL pipette (Eppendorf, catalog number: EP3123000047) or equivalent

28. Research Plus 100–1,000 μL pipette (Eppendorf, catalog number: EP3123000063) or equivalent

29. (Optional) Elite Variable Volume, multichannel pipette 10–100 μL (Thermo Fisher Scientific, Fisher Scientific, Fisherbrand, catalog number: 11825772)

30. Ultrasonic processor UP400S (400 W, 24 kHz) with Sonotrode H7 or H14 (Hielscher Ultrasonics)

31. Diaphragm Vacuum Pump LABOPORT (KNF, type: N 810.3 FT.18) or equivalent for filter sterilization

32. End-over-end rotator (any model)

33. Multiskan FC Microplate Photometer (Thermo Fisher Scientific, Thermo Scientific, catalog number: 51119000) or equivalent

34. Denovix QFX or equivalent fluorophore measuring device

35. 744 pH Meter (Metrohm, model: 744) or equivalent

36. Vortexer (any model)

37. Isotemp RT Advanced Hotplate Stirrer, 350 °C, Ceramic, Aluminum (Thermo Fisher Scientific, Fisher Scientific, Fisherbrand, catalog number: 15326607)

38. Spectrophotometer (any)

## Procedure

*Note: To maintain sample integrity, two dedicated sets of pipettes were utilized: one for bacterial cultures and a separate set for buffer preparation, protein purification, and RNase-free work. The clean pipette set was decontaminated every three months. This rigorous compartmentalization and maintenance schedule successfully prevented cross-contamination, precluding the need for filter tips.*

**A. MarathonRT expression in Rosetta 2(DE3)pLysS**

**A1. (Day 1) Transformation of MarathonRT plasmid into *E. coli* expression strain Rosetta 2(DE3)pLysS**

1. Transform the pLJSRSF7-T7-6xHis-SUMO-MarathonRT-CBD plasmid into Rosetta 2(DE3)pLysS chemically competent cells.

*Note: We recommend starting each protein expression batch with a fresh transformation reaction. Using more than 3-day-old transformants or storing the transformed bacteria in a glycerol stock may lead to mutations in the coding gene.*

a. Thaw 50 μL of competent cells on ice.

b. Add 100 ng of plasmid DNA to the competent cells and flick the tube a few times.

c. Return the tubes on ice for 30 min.

d. Heat-shock the cells for 20 s in a heat block at exactly 42 °C.

e. Place the tubes on ice for 2 min.

f. Add 450 μL of room temperature LB Miller broth (Recipe 22a) to the transformation reaction.

g. Incubate at 37 °C and 200–600 rpm for 60 min.

*Note: We recommend using an agitation speed of 200 rpm for orbital platform shakers, whereas 300–600 rpm is suitable when using a ThermoMixer.*

h. Plate 25, 50, and 100 μL of the transformation reaction onto an LB Miller agar plate (Recipe 22b) containing 50 μg/mL kanamycin (Recipe 1) and 35 μg/mL chloramphenicol (Recipe 2) and spread using sterile glass beads or a cell spreader.

i. Invert the plates and incubate at 37 °C for 12–16 h.

**A2. (Day 2) Starter culture for large-scale expression**

1. Transfer the plates with transformants to 4 °C.

**Pause point:** The transformation plates can be kept up to 3 days at 4 °C.

2. Inoculate and grow the overnight starter culture.

a. Pick a colony using a sterile inoculation loop and use it to inoculate 30 mL of LB broth containing 50 μg/mL kanamycin and 35 μg/mL chloramphenicol in a 250 mL Erlenmeyer flask.

b. Grow overnight in a shaking incubator at 37 °C and ~200 rpm for 12–16 h.

**A3. (Day 3) Large-scale expression of MarathonRT**

1. Measure the overnight culture optical density (OD_600_) from 10× dilution into LB broth.

2. Inoculate the large-scale culture by diluting the overnight starter culture to OD_600_ ~0.1 into 500 mL of LB broth (prewarmed to 37 °C) containing 50 μg/mL kanamycin and 35 μg/mL chloramphenicol in a 2 L Erlenmeyer flask.

*Note: We recommend using prewarmed LB broth. This significantly reduces the time required for the culture to reach an OD_600_ of 0.4–0.5.*

3. Grow the cells in a shaking incubator at 37 °C and ~200 rpm to OD_600_ 0.4–0.5.

*Note: Usually, it takes 1.5–3 h for the cells to reach the desired density.*

**Critical:** We noticed that growing the cells to a higher OD_600_ before the induction of protein expression decreases the yield significantly due to precipitation during purification.

4. Induce the culture by adding 250 μL of 1 M IPTG (Recipe 3) into 500 mL culture, which results in an IPTG final concentration of 0.5 mM.

5. Incubate the culture in a shaking incubator at 16 °C and ~200 rpm for 18–19 h.

**A4. (Day 4) Harvesting of the cells**

**Figure 1.**
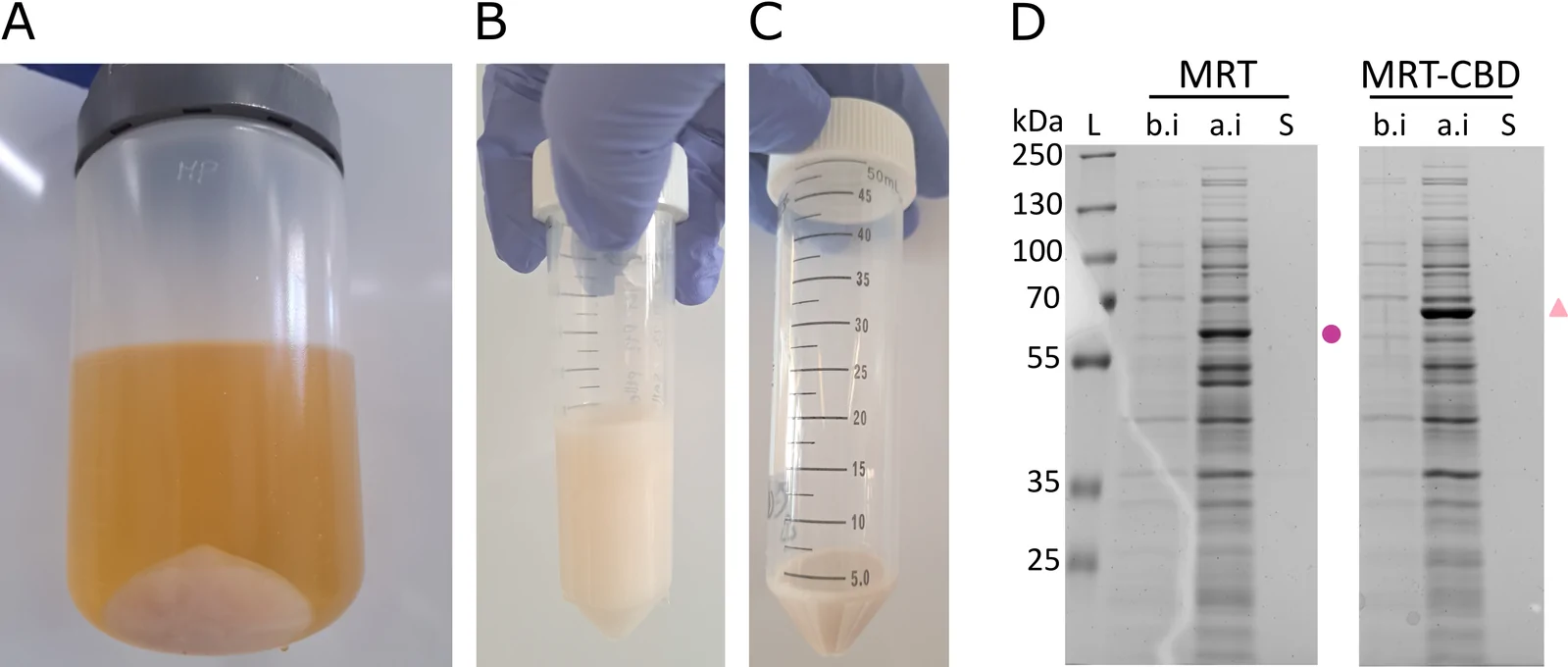
MarathonRT expression in *E. coli* Rosetta 2(DE3)pLysS cells. (A) Pelleted cells. (B) Washing the cells with 1× PBS. (C) Washed and pelleted cells. (D) MRT: MarathonRT; protein band is denoted with a purple circle; MRT-CBD: re-cloned MarathonRT with added C-terminal CBD-tag; protein band is denoted with a pink triangle; L: PageRuler Plus prestained protein ladder, 10–250 kDa; b.i.: sample taken from the bacterial culture before expression induction with IPTG; a.i.: sample taken from the bacterial culture after expressing the protein for 18 h at 16 °C; S: sample from supernatant after pelleting the cells.

1. Transfer the cells into a 1 L high-speed centrifugation bottle. Alternatively, we have also used 2 × 500 mL high-speed centrifugation bottles.

2. Harvest the cells by centrifugation at 5,000× *g* for 15 min at 4 °C (Figure 1A).

3. Carefully remove and discard the supernatant by pouring it over the pellet.

4. Resuspend the cells in 20 mL of 1× PBS (Recipe 22d) and transfer to a 50 mL centrifuge tube (Figure 1B).

5. Pellet the cells by centrifugation at 3,220× *g* for 10 min at 4 °C.

6. Carefully remove and discard the supernatant by pouring over the pellet (Figure 1C).

*Note: We recommend using a swing-bucket rotor for this step.*

**Pause point:** Washed cells can be stored at -70 °C for up to 1 week.

**Critical:** Storing the cells for more than 1 week at -70 °C may result in a degraded product.

**Critical:** Not washing the cells with 1× PBS might result in protein degradation and precipitation.

**Optional:** Users may add protease inhibitors to prevent further protein degradation and to increase the yield.

**B. One-step MarathonRT purification**

**(Day 5) Lysis of the cell pellet, on-column His-tag purification, and concentration of the protein**

**Critical:** Always keep the cell pellet on ice and use buffers stored on ice or at 4 °C.

**Critical:** Use nuclease-free laboratory supplies, reagents, and water.

1. Resuspend the cell pellet from 500 mL of culture in 20 mL of Buffer A (Recipe 23a) using a 25 mL serological pipette.

*Note: If the pellet was frozen, thaw the pellet on ice.*

2. Lyse the resuspended cells using a sonicator.

a. Set amplitude to 60% and cycle to 0.5 and use tip H7 or H14 (see Equipment).

b. Place the 50 mL centrifuge tube on ice and sonicate 5 × 30 s with 30 s rest time between each cycle. Alternatively, break the cells using a French press with 8,000 psi × 3 cycles to break the cells and genomic DNA.

*Note: Most DNases do not work at high salt concentrations. Hence, we have found sonication alone to be sufficient for the fragmentation of genomic DNA.*

**Figure 2.**
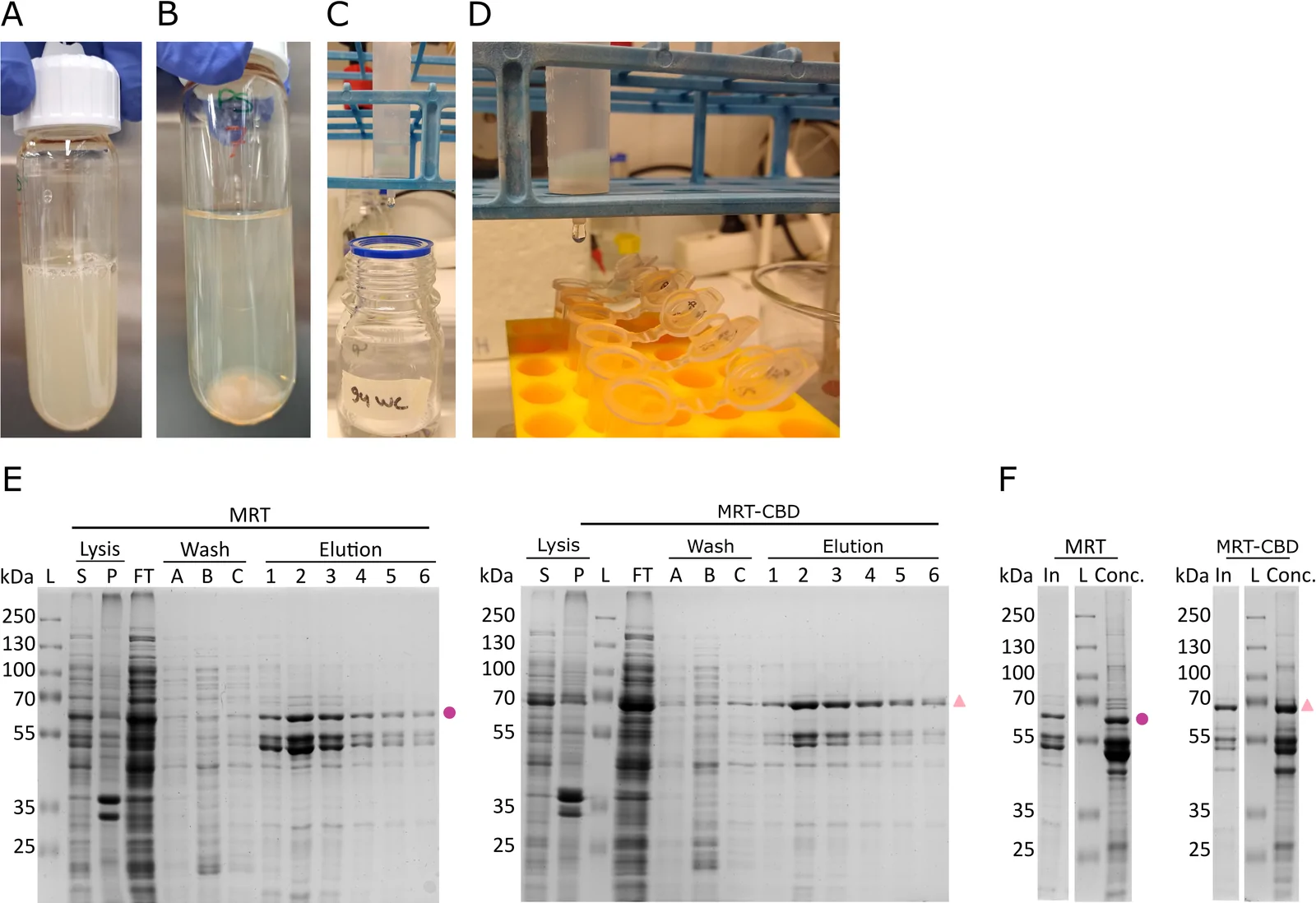
Cell lysis and affinity purification of MarathonRT. (A) Lysed cells. (B) Lysate after pelleting the cell debris. (C) Setup for washing the resin after binding MarathonRT to the HisPur Ni-NTA resin. (D) Collection of elution fractions. (E, F) Collected samples analyzed on 10% SDS-PAGE. L: PageRuler Plus prestained protein ladder, 10–250 kDa. Full-length protein product indicated as follows: on the left panel, MRT (dot); on the right panel, MRT-CBD (triangle). (E) Lysis and affinity purification. S: sample from the supernatant after clarifying the lysate from the cell debris; P: sample from the cell debris pellet after clarifying the lysate; FT: flowthrough from the column after loading the sample; A–C: samples taken after running wash buffers through the column; 1–6: collected elution fractions. (F) Concentrated protein samples. In: pooled elution fractions; Conc: concentrated protein sample.

3. Transfer the lysed cells into a 50 mL high-speed centrifuge tube (Figure 2A).

4. Clarify the lysate in a high-speed centrifuge at 22,000× *g* for 45 min at 4 °C (Figure 2B).

*Note: In the original publication [13], an ultracentrifuge at 30,000×* g *for 30 min at 4 °C was used; in [9], authors used 13,000×* g *for 30 min at 4 °C. We did not test these conditions.*

5. During centrifugation, equilibrate His-Pur Ni-NTA resin:

a. Carefully transfer 2 mL of the slurry (50% beads–50% buffer) into a 15 mL centrifuge tube.

b. Centrifuge the slurry at 700× *g* for 2 min at 4 °C.

*Note: We recommend using a swing-bucket rotor.*

c. Carefully remove and discard the storage buffer.

d. Add 10 mL of buffer A (Recipe 23a) to the resin, then mix until the resin is fully suspended.

e. Centrifuge the slurry at 700× *g* for 2 min at 4 °C.

f. Carefully remove and discard the buffer.

g. Keep the equilibrated resin on ice.

6. After centrifugation, pour the supernatant into a 150 or 500 mL 0.2 μm upper-cup filter on top of a 100 mL pre-cooled bottle and vacuum-filtrate the cleared lysate.

*Note: Using an upper-cup filter with a bigger surface area (500 mL) speeds up the filtration process, especially if the sample is still a little viscous from the genomic DNA. See Troubleshooting.*

7. Transfer the filtered lysate and equilibrated resin from step B5 into a 50 mL centrifugation tube.

8. Mix on an end-over-end rotator for 1 h at 4 °C for batch binding.

**Critical:** Keep the rotation speed low to avoid foaming of the sample.

9. During the mixing, prepare the setup for the gravity flow affinity purification (Figure 2).

a. Place a plastic 50 mL centrifuge tube holder perpendicularly on top of two tube holders.

b. Place the empty 10 mL self-packable column into the 50 mL centrifuge tube holder.

c. Cool down 4× 100 mL glass bottles to 4 °C for collecting the flowthrough and wash samples A, B, and C.

d. Cool down 7 microfuge tubes to 4 °C for collecting elution samples.

10. Pour the resin–protein mix into the 10 mL self-packable gravity-flow column and allow the flowthrough to drain from the resin by gravity flow.

*Note: To minimize product loss, rinse the 50 mL centrifuge tube with 1–5 mL of Buffer A (Recipe 23a) to ensure maximum resin recovery, and transfer it to the self-packed column.*

11. Collect the flowthrough into the pre-cooled 100 mL glass bottle.

12. Wash the resin with:

a. 20 mL of Buffer A (Recipe 23a)

b. 20 mL of Buffer B (Recipe 23b)

c. 20 mL of Buffer C (Recipe 23c) (Figure 2C)

**Critical:** Add the next buffer to the column only once the previous buffer has fully entered the packed resin bed. However, do not let the column dry.

**Optional:** Collect the wash steps for downstream analysis to check that the protein is not lost during the wash steps.

*Note: We recommend recording the time it takes to perform the affinity purification every time. Significantly slower gravity flow times are an indication that the column is clogged, and the protein has precipitated. See Troubleshooting.*

13. Elute the protein with 10 mL of Buffer D (Recipe 23d) and collect 6 × 1 mL fractions into pre-cooled 1.5 mL microfuge tubes (Figure 2D).

**Pause point:** Eluted samples can be kept at 4 °C overnight. Keeping the protein longer in a high concentration of imidazole might damage the protein.

**Optional:** Centrifuge the eluted samples at 10,000× *g* for 10 min at 4 °C. This ensures that none of the protein is precipitated. If a white pellet appears, collect the supernatant only and continue with the next step. There is a risk that you have lost most of the protein if a pellet appears. See Troubleshooting.

**Optional:** Although we used self-packed Ni-NTA resin for MRT one-step purification, we recommend using a pre-packed column and a peristaltic pump (or ÄKTA FPLC) as an alternative. However, for optimization purposes, it is practical to use a system where precipitation is easily detectable and cheap to operate. Using gravity flow enables visual confirmation of at which step and time point precipitation might occur.

14. Analyze the collected fractions on 10% SDS-PAGE (Figure 2E).

*Note: MRT is 62 kDa and MRT-CBD is 68 kDa in size. Typically, we load 1 μL of each collected elution fraction onto a 1.5 mm SDS-PAGE gel.*

15. Pool the fractions containing MarathonRT into a 15 mL centrifuge tube using a 1 mL pipette.

16. Exchange the buffer and concentrate the sample using an Amicon Ultra Centrifugal Filter, 50 kDa molecular weight cutoff (MWCO). All centrifugation steps are performed using a swing-bucket rotor.

*Note: The user can opt for a higher MWCO concentrator. This will yield a purer protein preparation, as lower molecular weight protein contaminants are more efficiently removed. However, care must be taken not to lose the target protein in the flowthrough and/or excessively sacrificing the yield.*

**Critical:** Take care not to touch the concentrator membrane with the pipette tip. This could break the membrane, and the protein will end up in the flowthrough. See Troubleshooting.

**Critical:** Using any other concentrator with a smaller membrane area will result in at least 2× longer time spent on the concentration of the protein.

a. Transfer the pooled fractions into the concentrator.

b. Centrifuge at 3,220× *g* for 10 min at 4 °C.

c. Carefully mix the sample up and down using a 1 mL pipette to avoid the formation of a strong glycerol gradient that will slow down the concentration significantly.

d. Repeat steps B16b–c until there is 500–1,000 μL of sample left in the concentrator.

**Optional:** Collect the flowthrough for SDS-PAGE analysis. There should be no MarathonRT in the flowthrough sample. See Troubleshooting.

e. Add Buffer G (-glycerol) (Recipe 23e) into the concentrator up to 15 mL and mix by pipetting up and down.

f. Repeat steps B16b–c until there is 500–1,000 μL sample left in the concentrator.

**Optional:** Collect the flowthrough for SDS-PAGE analysis. There should be no protein in the flowthrough sample. See Troubleshooting.

g. Add Buffer G (-glycerol) (Recipe 23e) into the concentrator up to 15 mL and mix by pipetting up and down.

h. Repeat steps B16b–c until the sample is concentrated down to the 500 μL line.

*Note: On these specific concentrators, if the bottom concave of the liquid meniscus is at the 500 μL line, then the total retrieved volume is 533 μL.*

i. Carefully transfer the concentrated protein sample to a clean 1.5 mL microfuge tube.

j. Add ~667 μL of sterile cold 90% glycerol (final volume 1,200 μL) and mix by pipetting up and down.

**Critical:** Take time to mix the glycerol with the concentrated protein properly. Otherwise, when aliquoted, the samples will have a varying protein concentration. Avoid bubbles!

k. Analyze the concentrated protein sample on 10% SDS-PAGE (Figure 2F).

*Note: Typically, we have loaded 1–5 μL of the concentrated protein per lane on a 1.5 mm SDS-PAGE.*

17. Aliquot the protein sample into ~12 microfuge tubes and store at -70 °C (long-term) or -20 °C (short-term).

*Notes:*

*1. The protein samples can be stored at -20 °C for up to 8 weeks with no significant decrease in activity and at -70 °C for at least a year with no significant change in the activity [1].*

*2. We recommend flash-freezing the protein sample for storage at -70 °C.*

**C. RNA template production for MarathonRT unit definition**

**(Day 6) Producing a standardized RNA template for reverse transcription reaction**

1. Linearize 10 μg of the plasmid for RNA template generation (see Biological materials) using *Eco*RI for run-off transcription reaction.

a. Assemble the linearization reaction at room temperature in a 1.5 mL microfuge tube as shown in Table 1.

**Table 1.** Pipetting table for plasmid linearization

**Component**	**Final concentration**	**1**× **reaction**
Plasmid, 10 μg	N/D	X μL
*Eco*RI	N/D	2 μL
10× Fast Digest Buffer	1×	4 μL
Nuclease-free water	N/A	34 μL–X μL
Total volume	N/A	40 μL

b. Mix by pipetting up and down and pulse-spin in a centrifuge to collect the liquid to the bottom of the microfuge tube.

c. Incubate the reaction for 1 h at 37 °C.

d. Dephosphorylate the digested plasmid by adding 1 μL of FastAP. Mix by pipetting up and down.

e. Incubate for 30 min at 37 °C.

f. Incubate the reaction for 5 min at 80 °C to inactivate the enzyme.

**Optional:** After this step, you can check the linearization efficiency by electrophoresis on 1% agarose gel. The linearized DNA should be clearly visible at 4,331 bp as a single band.

2. Purify the linearized plasmid using NucleoSpin Gel and PCR Clean-up kit. Follow the manufacturer’s protocol.

**Critical:** Elute in nuclease-free water.

**Alternative:** In principle, any PCR purification kit with an appropriate cutoff size should work for this purification step.

3. Measure DNA concentration on a NanoDrop or an equivalent UV spectrophotometer.

4. Assemble the in vitro transcription reaction at room temperature in a 1.5 mL microfuge tube as shown in Table 2.

**Table 2.** Pipetting table for in vitro transcription reaction

Component	Final concentration	1× reaction
Linearized plasmid from step C2, 1 μg	N/D	X μL
5× transcription buffer	1×	10 μL
NTPs, 10 mM each	2 mM each	10 μL
RNAsin Plus, 40 U/μL	0.8 U	1 μL
T7 RNA polymerase 20 U μL	30 U	1.5 μL
Nuclease-free water	N/A	27.5 μL–X μL
Final volume	N/A	50 μL

5. Incubate the reaction for a minimum of 2 h at 37 °C.

*Note: The reaction should become milky due to inorganic phosphate (PP_i_) precipitation. Typically, we incubate the reaction for 3 h.*

6. Degrade the DNA template by adding 2 μL of RQ1 DNase. Mix by pipetting up and down.

7. Incubate for 15–20 min at 37 °C.

8. Centrifuge the sample at 16,000× *g* for 5 min at room temperature.

*Note: This centrifugation step helps to remove the precipitated inorganic phosphate that can be seen as a small white pellet. However, it may not always be visible.*

9. Transfer the supernatant into a clean 1.5 mL centrifuge tube.

**Pause point:** The in vitro transcription (IVT) reaction can be stored at -70 °C for up to 3 days.

*Note: We recommend analyzing the IVT reaction by electrophoresis on a 5% denaturing urea-polyacrylamide gel. The synthesized 642 nt RNA should be clearly visible. In our experience, loading 1/100^th^ volume of the reaction is sufficient to visualize the synthesized RNA.*

**Figure 3.**
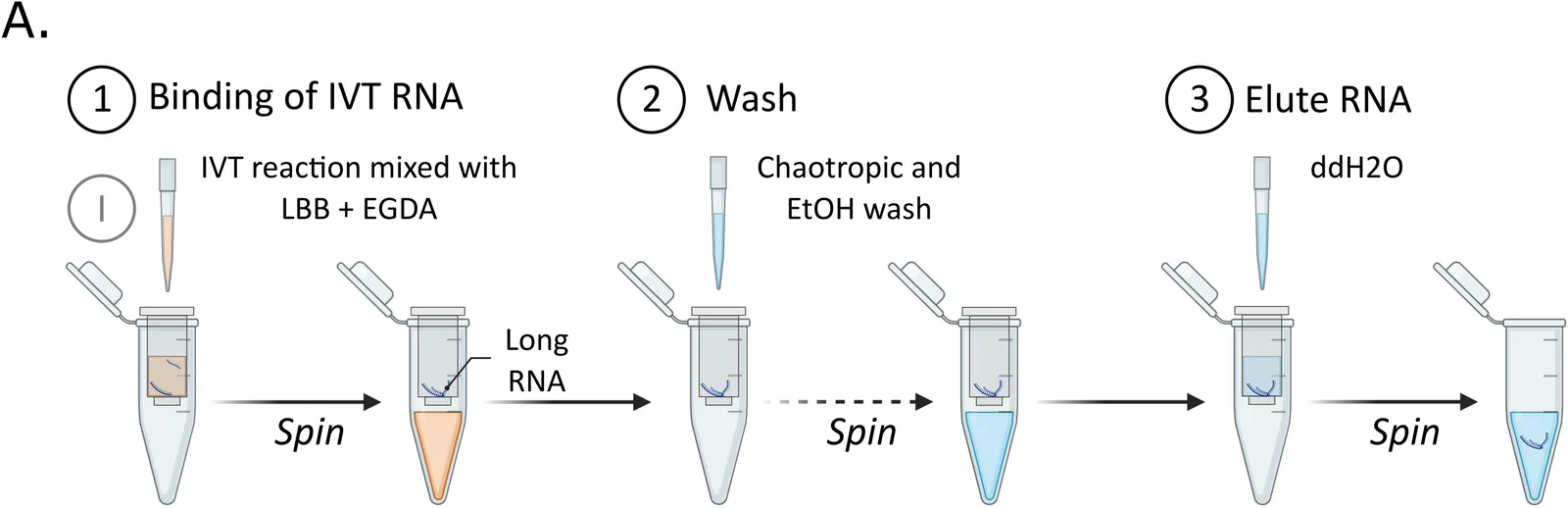
Schematic overview of in vitro–transcribed (IVT) RNA column purification. (1) In vitro–transcribed RNA sample is mixed with long RNA binding buffer (LBB) and EGDA to facilitate the RNA binding to the silica matrix. (2) Bound RNA is washed with a chaotropic wash (CW) and ethanol wash (EW). (3) RNA is eluted with ultra-pure water.

10. Purify the synthesized RNA using NucleoSpin RNA columns and custom buffers [25] (Recipes 25a–c) following the scheme shown in Figure 3.

a. Adjust the volume of the IVT reaction with nuclease-free water to 100 μL.

b. Mix the sample with 250 μL of LBB (Recipe 25a).

c. Add 190 μL of EGDA and mix thoroughly by vortexing.

d. Load the sample into the column inserted in the collection tube.

e. Centrifuge the sample at 11,000× *g* for 1 min at room temperature.

f. Place the column into a new collection tube.

g. Wash the column with 500 μL of CW (Recipe 25b).

h. Centrifuge the sample at 11,000× *g* for 1 min at room temperature.

i. Discard the flowthrough (FT).

j. Wash the column with 500 μL of EW (Recipe 25c).

k. Centrifuge the sample at 11,000× *g* for 1 min at room temperature.

l. Discard the FT.

m. Repeat the EW wash.

n. Centrifuge the sample at 11,000× *g* for 2 min at room temperature to remove traces of EtOH.

o. Place the column into a clean 1.5 mL centrifuge tube.

p. Elute the RNA by carefully pipetting 50 μL of nuclease-free water at 50 °C in the middle of the silica membrane.

q. Centrifuge the sample at 11,000× *g* for 1 min at room temperature.

r. Measure RNA concentration using the Qubit RNA Quantitation kit on a DeNovix fluorometer (or a similar fluorometer).

11. Aliquot and store the purified synthetic RNA at -70 °C for up to a year. Avoid repeated freeze-thaw cycles, as this will result in degraded RNA.

*Notes:*

*1. We recommend analyzing the purified RNA by electrophoresis on 5% denaturing urea-polyacrylamide gel. The synthesized 642 nt RNA should be clearly visible. In our experience, loading 20–50 ng is sufficient to visualize the synthesized RNA.*

*2. See Troubleshooting if the user experiences problems with the RNA yield.*

**D. Determining MarathonRT concentration using Bradford assay**

**(Day 7) Absolute quantification of protein yield**

*Note: All protein dilutions are measured in triplicate.*

1. Prepare BSA dilution to 0.1 μg/μL for the standard curve (Recipes 18 and 19).

2. Pipette the diluted BSA onto the microplate in different concentrations as shown in Table 3.

**Table 3.** Pipetting scheme for BSA dilutions for the standard curve

Row on the plate	BSA total amount	BSA concentration	0.1 μg/μL BSA	Nuclease-free H_2_O
A	0 μg	0 μg/μL	0 μL	100 μL
B	0 μg	0 μg/μL	0 μL	100 μL
C	0.5 μg	0.005 μg/μL	5 μL	95 μL
D	1 μg	0.01 μg/μL	10 μL	90 μL
E	2 μg	0.02 μg/μL	30 μL	70 μL
F	3 μg	0.03 μg/μL	50 μL	50 μL
G	5 μg	0.05 μg/μL	80 μL	20 μL
H	10 μg	0.1 μg/μL	100 μL	0 μL

3. Prepare dilutions of the concentrated protein to fit the linear range of the standard curve.

a. For a 500× dilution, pipette 998 μL of nuclease-free water into a 1.5 mL microfuge tube and add 2 μL of the protein stock.

b. Mix by pipetting up and down.

c. Pipette 100 μL onto the microplate in triplicate.

d. For a 1,000× dilution, pipette 50 μL of 500× diluted protein directly onto the microplate in triplicate and add 50 μL of nuclease-free water.

*Note: The absolute concentration of protein measurable with this assay setup is 0.1 μg/μL. If the protein concentration is out of the linear range of the assay, a different dilution is needed. We recommend having 2–3 different dilutions to start with, e.g., 300×, 500×, and 1,000×, to measure the protein concentration.*

**Critical:** High salinity buffers will interfere with the measurements. Do not use buffer for the dilution!

4. Pipette 200 μL of nuclease-free water as the blank sample onto the microplate row A in triplicate.

5. Pipette 100 μL of Bradford reagent (Recipe 24a) into all samples, except into the blank samples in row A.

*Note: We recommend using a multichannel pipette for this step.*

6. Mix the samples by pipetting up and down.

**Critical:** Avoid bubbles! If there are bubbles in the wells, use, for example, a syringe needle or a clean pipette tip to pop the bubbles. The bubbles will interfere with the plate-reader measurements.

7. Incubate the microplate for at least 5 min and no more than 60 min at room temperature for the color to develop.

*Note: We recommend keeping the incubation time the same for each measurement.*

8. Measure absorbance at 595 and 450 nm.

*Note: We recommend, if possible, creating a program in the plate reader for the measurement and automatic subtraction of the blank readings.*

9. Draw the standard curve by dividing the net absorbance values at 595 and 450 nm (see Data analysis).

*Note: Subtract the blank wells (no dye, row A) if this is not programmed to be done automatically by the plate reader. The 0 μg BSA (dye only, row B) value should be included as a data point.*

10. Calculate the protein amount in your sample based on linear regression of the calibration curve (see Data analysis).

*Note: See Troubleshooting if the results are inconsistent.*

**E. MarathonRT unit definition**

**(Day 7) Measuring the activity of the purified enzyme batch**

**MarathonRT unit definition:** 1 unit of MarathonRT is defined as amount of enzyme that can release 1 nmol of inorganic pyrophosphate (PP_i_) in 30 min at 42 °C, when using RNA template [produced from #101156 plasmid (Addgene) linearized with *Eco*RI] as a template and gene-specific reverse transcription primer (5′-TCACTGCATACGACGATTCTG-3′) in the following reaction conditions: 50 mM Tris-HCl pH 8.3, 200 mM KCl, 2 mM MgCl_2_, 5 mM DTT, 20% glycerol, 1 U/μL RNasin Plus RNase inhibitor, 0.5 mM (each) dNTPs, 0.05 μm gene-specific primer, 300 ng RNA template.

*Note: This section provides a detailed protocol on how to define MarathonRT units using a colorimetric assay. This colorimetric assay estimates the enzyme activity based on the pyrophosphate (PP_i_) released during the enzymatic reaction. PP_i_ can then be detected by a coupled reaction using inorganic pyrophosphatase (PPase). The reaction with PPase produces inorganic phosphate (Pi), which subsequently reacts with molybdate and forms phosphomolybdate. Phosphomolybdate forms a complex with malachite green, which strongly absorbs at 620–650 nm* [26,27]. *In addition, this assay enables the user to measure the activity of different enzyme batches in a comparable manner.*

**Critical:** All reagents and solutions must be prepared and stored in clean plastic containers unless stated otherwise. Having any component used in this assay in glassware (including nuclease-free water) risks phosphate contamination and skewing of the results. Do not use autoclaved pipette tips.

1. Prepare phosphate (P_i_) calibration/standard curve (Recipe 26b).

*Note: The standard curve must be prepared prior to determining the enzyme activity. A fresh standard curve must be done if a fresh batch of reagents is used.*

a. Prepare serial dilutions of the P_i_ stock (Recipe 21a) to yield 50 μm.

**Critical:** Always prepare fresh dilutions when repeating the calibration!

i. Prepare 1 mM P_i_ by pipetting 990 μL of nuclease-free water into a 1.5 mL microfuge tube and adding 10 μL of 100 mM P_i_. Mix by vortex and pulse-spin.

ii. Prepare 50 μm P_i_ by pipetting 950 μL of nuclease-free water into a 1.5 mL microfuge tube and adding 50 μL of 1 mM P_i_ from the previous step. Mix by vortex and pulse-spin.

b. Prepare standard curve dilutions of the freshly prepared 50 μm P_i_ directly to the microplate as shown in Recipe 26b.

**Critical:** Prepare all dilutions in triplicate.

c. Prepare the detection solution (DS) (Recipe 26c) freshly in a 1.5 mL or 2 mL microfuge tube.

*Note: We recommend preparing the DS for 100 reactions. This allows for the use of a multichannel pipette. Use on the same day.*

d. Pipette 20 μL of DS solution to the P_i_ dilutions on the microplate. Mix by pipetting up and down.

*Note: We recommend using a multichannel pipette.*

**Critical:** Avoid bubbles! If there are bubbles in the wells, use, for example, a syringe needle to pop the bubbles. The bubbles will interfere with the plate-reader measurements.

e. Incubate for 30 min at room temperature to develop the color.

f. Measure the absorbance at 620 nm using a plate reader.

g. Export the values and analyze the data (see Data analysis).

2. Measure the PP_i_ produced by MarathonRT during the RT reaction.

**Critical:** Perform the calibration and reverse transcription reaction using the same DS solution. Typically, we perform the standard curve P_i _and reverse transcription reaction PP_i_ measurements on the same microplate.

a. Dilute MarathonRT protein stock to 0.6 μg/μL using MarathonRT storage buffer/Buffer G (+glycerol) (Recipe 23f) into a 1.5 mL microfuge tube.

i. Use the protein concentration obtained from Bradford Assay (see step D10) to calculate the necessary dilution, as shown in Table 4 with the typical yield as an example.

**Table 4.** MarathonRT dilution to 0.6 μg/μL with the total volume of 12 μL

Enzyme	Stock concentration (μg/μL)	Dilution factor	MarathonRT stock (μL)	Storage buffer (μL)
MarathonRT	4.4 μg/μL	4.4/0.6 = 7.3×	12/7.3 = 1.6	12-1.6 = 10.4

b. Follow the MarathonRT activity assay reaction setup in Table 5.

**Table 5.** MarathonRT activity assay reaction setup. Prepare the reverse transcription reaction in duplicate or triplicate in a 200 μL PCR tube.

Reagent	Final concentration	Amount per 1 reaction
RNA template*	300 ng	X
RT primer (1 μm)	0.05 μm	1 μL
Nuclease-free water	N.A.	Up to 7 μL
1. Mix RNA template, primer, nuclease-free water, and pulse-spin. 2. Incubate for 30 s at 95 °C in a thermocycler. 3. Anneal the primer to the template by incubating the reaction at room temperature for 5 min. 4. Add the components listed below. **Critical:** Add the MarathonRT as last. 5. Mix well and pulse-spin.		
MRT 2× reaction buffer**	1×	10 μL
dNTPs (10 mM)	0.5 mM each	1 μL
PPase (0.1 U/ μL)	1 mU/μL	0.5 μL
RNAsin Plus (40 U/μL)	1 U/μL	0.5 μL
MarathonRT (unknown activity)	N.A.	1 μL
Total volume		20 μL

* From step C11.

**Can be substituted with 2× reaction buffer with 4 mM MgCl_2_, as long as all the assays are done with the same buffer.

*Note: We recommend preparing a master mix for both steps, mixing well, and pulse-spinning. Usually, we prepare a control where there is 1 μL of nuclease-free water instead of MarathonRT.*

c. Incubate the reactions for 1 h at 42 °C in a thermocycler.

d. After the incubation, transfer the tubes to ice.

e. Add 60 μL of nuclease-free water to the reactions to yield 80 μL in total for each reaction.

f. Vortex and pulse-spin.

g. Prepare a 4× dilution directly onto the microplate from each diluted reverse transcription reaction in triplicate by pipetting 20 μL of sample from step E2e and adding 60 μL of nuclease-free water.

**Figure 4.**
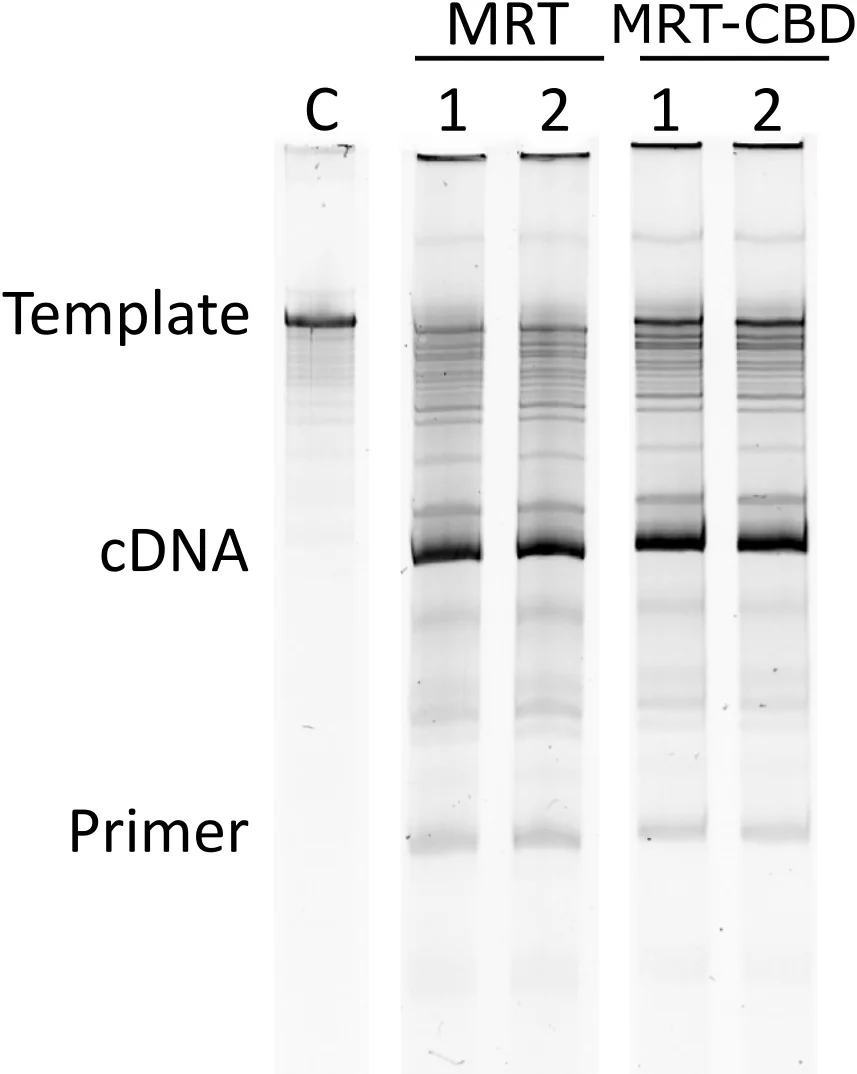
Column-purified RNA template and analysis of activity assay on 6% urea-PAA. C: column-purified IVT produced RNA template (642 nt); 1, 2: technical duplicates of RT reactions; cDNA-277 nt, primer: RT primer 21 nt.

*Note: This will leave ~20 μL of reverse transcription reaction that can be analyzed on 10% urea-PAA (Figure 4).*

**Critical:** Prepare all dilutions in triplicate.

*Note: If the protein sample is less concentrated or more concentrated and the reverse transcription yield is lower or higher, a different dilution of the reverse transcription reaction can be used.*

h. Pipette 20 μL of DS solution into the samples on the microplate.

i. Mix by pipetting up and down.

j. Incubate for 30 min at room temperature to develop the color.

**Critical:** Avoid bubbles! If there are bubbles in the wells, use, for example, a syringe needle to pop the bubbles. The bubbles will interfere with the plate-reader measurements.

k. Measure the absorbance at 620 nm using a plate reader.

l. Export the values and analyze the data (see Data analysis).

## Data analysis

Protein concentration was determined by Bradford assay following the calculations shown in [1] Supp. File S2.

Enzyme activity was determined by colorimetric assay based on pyrophosphate released during the RT reaction, following the calculations shown in [1] Supp. Files S3, S5, and S6.

All experiments were conducted at least three times unless otherwise specified.

## Validation of protocol

This protocol has been used and validated in the following research article:

- Pedor et al. [1]. Production of MarathonRT and its comparison with commercial reverse transcriptases for tRNA sequencing library preparation. *bioRxiv.* [Figure 1, Figure 3, Supp. Figure S2, Supp. File S2 (Bradford assay calculations), Supp. File S3 (storability measurements), Supp. File S5 (resistance to freeze-thaw cycling over-time), Supp. File S6 (storability of diluted preparation). Supp. File S7 (optimal enzyme to template ratio)].

## General notes and troubleshooting

**General notes**

1. Avoid foaming the protein-containing samples.

2. Always keep the cell pellet and protein-containing samples on ice or in the cold room.

3. Mix the samples well. This is especially important for Bradford assay and enzymatic activity measurements.

4. Be consistent. Use the same incubation times, pipettes, and instruments to get consistent results.

**Troubleshooting**

**Problem 1:** Occurrence of a white precipitate in protein-containing samples.

Possible causes:

a. Using transformants older than 3 days for expression.

b. Inducing the protein expression at OD_600_ > 0.5.

c. Wrong buffer pH or salt concentration.

d. Keeping the protein longer than overnight in a high concentration of imidazole.

e. Concentrating the protein into too low volume.

Solutions: If the protein has precipitated, throw away the sample and start again. For prevention:

a. Always use a fresh transformant.

b. Induce the protein expression at OD_600_ < 0.5.

c. Make sure to check the pH of your buffers.

d. Do not keep your protein for longer than 1 night in a high concentration of imidazole.

e. Aim to use the concentration guidelines written in the procedure.

**Problem 2:** Protein sample is too viscous after clearing the lysate and does not want to go through the filter.

Possible causes: Too short sonication time/too few cycles of sonication/too small probe.

Solution: Change the sonication parameters (increased time or bigger probe).

**Problem 3:** Protein purification on an affinity column is taking significantly longer than the norm.

Possible causes:

a. The protein has precipitated.

b. There is an air bubble blocking the outlet.

Solutions:

a. Discard the protein batch and start again.

b. Tap the column to get rid of the air bubble.

**Problem 4:** The protein sample leaked into the flowthrough collection compartment of the protein concentrator (detected on SDS-PAGE).

Possible causes:

a. You have pierced the membrane with the pipette tip, and the protein flew into the flowthrough compartment.

b. You have used a protein concentrator with too high MWCO.

c. The concentrator that you are using is of inconsistent quality.

Solution: If you recognize this problem on the same day as the concentration and have kept the flowthrough on ice, use a new concentrator and repeat the concentration step. Remember to consider the imidazole concentration in the current sample and estimate the dilution needed to get the imidazole concentration close to zero. If you recognize this problem on the next day, the protein has been too long in the imidazole. It is best to start again.

**Problem 5:** There is a significant variation in the replicas during assay (Bradford or enzyme activity) measurements.

Possible cause: The samples are not mixed properly.

Solution: Mix the samples by pipetting up and down at least 10× while drawing at least 90% of the sample volume into the pipette tip.

**Problem 6:** The RNA yield from column purification is lower than expected.

Possible causes:

a. Mistake in buffer preparation.

b. Problem with in vitro transcription reaction.

Solutions:

a. Prepare new buffers and analyze the flowthrough from wash steps by electrophoresis on a 5% denaturing urea-polyacrylamide gel. This will help to assess the binding efficiency of the column. Prior to analysis, precipitate the wash step flowthrough with ethanol or isopropanol.

b. Try longer in vitro transcription reaction time, 500 ng or 1,500 ng of template DNA instead of 1,000 ng. It might help to consult the troubleshooting guide for the T7 polymerase.
